# Examining the Effect of Household Wealth and Migration Status on Safe Delivery Care in Urban India, 1992–2006

**DOI:** 10.1371/journal.pone.0044901

**Published:** 2012-09-07

**Authors:** Prashant Kumar Singh, Rajesh Kumar Rai, Lucky Singh

**Affiliations:** 1 International Institute for Population Sciences, Mumbai, Maharashtra, India; 2 Tata Institute of Social Sciences, Mumbai, Maharashtra, India; Kenya Medical Research Institute - Wellcome Trust Research Programme, Kenya

## Abstract

**Background:**

Although the urban health issue has been of long-standing interest to public health researchers, majority of the studies have looked upon the urban poor and migrants as distinct subgroups. Another concern is, whether being poor and at the same time migrant leads to a double disadvantage in the utilization of maternal health services? This study aims to examine the trends and factors that affect safe delivery care utilization among the migrants and the poor in urban India.

**Methodology/Principal Findings:**

*Using data from the National Family Health Survey, 1992–93 and 2005–06, this study grouped the household wealth and migration status into four distinct categories* poor-migrant, poor-non migrant, non poor-migrant, non poor-non migrant. Both chi-square test and binary logistic regression were performed to examine the influence of household wealth and migration status on safe delivery care utilization among women who had experienced a birth in the four years preceding the survey. Results suggest a decline in safe delivery care among poor-migrant women during 1992–2006**.** The present study identifies two distinct groups in terms of safe delivery care utilization in urban India – one for poor-migrant and one for non poor-non migrants. While poor-migrant women were most vulnerable, non poor-non migrant women were the highest users of safe delivery care.

**Conclusion:**

This study reiterates the inequality that underlies the utilization of maternal healthcare services not only by the urban poor but also by poor-migrant women, who deserve special attention. The ongoing programmatic efforts under the National Urban Health Mission should start focusing on the poorest of the poor groups such as poor-migrant women. Importantly, there should be continuous evaluation to examine the progress among target groups within urban areas.

## Introduction

Maternal health remains a major challenge to the health system in developing countries and countries in transition. Global leaders were deeply concerned with the reduction in the Maternal Mortality Ratio (MMR) and they signed the Safe Motherhood Initiative in 1987. The International Conference in Population and Development (ICPD) held in 1994 also focused on this. Global action on the reduction of MMR was restated and placed as the fifth target of the United Nations Millennium Development Goals (MDG-5) in 2000. Despite considerable socioeconomic development and improvement in the utilization of maternal health services, recent estimates show that there has been insufficient progress in reducing maternal mortality in India [1], [2]. In last two decades, the MMR (defined as the number of maternal deaths during a given time period per 100,000 live births during the same time-period) in India has declined from 600 in 1990 to 200 in 2010 [2]. However, of the total global maternal deaths – estimated at 287000 in 2010– India alone contributes 19% (56000) [2].

National reporting of health indicators focuses on sub-national averages. However, data on the distribution of health services within the country and between population subgroups are equally important [3]. Such data facilitate the identification of health inequities – unfair and unavoidable differences in the provision of health services – that arise for example from socioeconomic status, geographical location and so on [3]. One of the most common findings in developing countries regarding the utilization of health services has been the advantage of residing in urban areas over rural areas [4]–[11]. The differences in maternal mortality between urban and rural areas within poor countries are substantial, irrespective of world regions [12]. The urban health advantage has often been attributed to the improved modern health care system that facilitates public health interventions [13], [14]. Additionally, urban areas offer more choices, ranging from greater availability of food, housing and health services to employment opportunities [15]. Improved electricity, transportation, water and sanitation services are also, on average, more widely available in urban areas than in rural [16], [17]. Factors that determine health status differ between urban and rural areas, given the differences in environment and in households, as well as individual opportunities and choices [18].

The urban advantage in health care service utilization has apparently faded in recent decades, since the urban population explosion in most of the developing countries including India has not been matched with an adequate expansion of sanitation, health services and livelihood opportunities [19], [20]. According to the projections of the United Nations Population Division, two-thirds of the global population is likely to be urban by 2050. The world’s population, as a whole, is expected to grow by 2.5 billion from 2007 to 2050, with the cities and towns of developing countries absorbing most of this additional population [21]. During the last few decades, India, like many other developing countries, has experienced rapid urbanization. According to the 2011 Census, 377 million people, roughly 31% of the total population were residing in urban areas [22]. It has been estimated that by 2020, over 40% of India’s population is expected to be urban [23], [24]. Despite India’s impressive economic performance over the past two decades, the number of urban poor has not reduced significantly [25]. Moreover, the ratio of urban poverty in some of the populous states is higher than that of rural poverty – leading to the phenomenon of “Urbanization of Poverty” in India [26]. On the other hand, the unprecedented growth of India’s urban population due to high migration exerts a great strain on the already overburdened health infrastructure and civic amenities [20], [27].

In recent years, the health status of the urban poor in developing countries has been documented in several studies [6], [18], [28]–[30]. Using Demographic and Health Survey datasets from ten developing countries, a study has shown that the socioeconomic gradient in childhood stunting is indeed higher in urban areas [29]. The same finding was also observed for Sub-Saharan Africa [13]. A few studies documented that the poor living in urban areas in India are at a disadvantage compared to the non-poor in the utilization of maternal and child health services [31]–[33]. A detailed study of the quality of care provided by private and public medical practitioners across seven neighbourhoods in Delhi, India, found that the urban poor receive low-quality care when compared to the urban non-poor [34]. Similarly, one year birth surveillance system covering a population over 280,000 in 48 vulnerable slum localities in Mumbai, India found that the poorer group had less advantageous demographic and environmental profiles, and indicators of compromised maternal and newborn health than the richer group [35]. An estimate presented by the Urban Health Resource Centre in 2006, suggests that about 79% of urban poor women deliver their children at home in the absence of trained professionals in the state of Rajasthan, India [36].

In public health literature, the “healthy migrant hypothesis” is frequently discussed, particularly in developed countries, which states that the migrants represent a selectively healthier group that is not representative of all potential migrants from origin societies [37]. As a result, their health advantage stands out when they are compared with the general population at destination [38]. Although, this theory has been inadequately tested, but few studies conducted in developed countries have shown that despite immigrants’ socioeconomic disadvantages, they are generally healthier than the native-born population [39], [40]. However, on the other hand, studies from developing countries have acknowledged adverse maternal, newborn and child health, and higher mortality rates among urban migrants compared to native urban dwellers or sometimes even their rural counterparts [41]–[46]. Using data from 17 countries, one study observed that the child survival prospects of rural to urban migrants were higher than those from a rural origin and lower than those of urban non-migrants [46]. A study from Bangladesh highlighted that inflation due to migration from rural to urban accelerates the under-five mortality among migrants in urban areas [47]. However, a study from India did not find any effect of rural to urban migration on child survival [48]. Some studies maintain that knowledge and awareness about key maternal health components were below average among migrants when compared to urban natives [49]. Also, migrants in India are generally drawn from less privileged sections and as such may have to face various hardships [50].

Recognizing urban health needs, the Government of India has launched several schemes and programs. These include the Urban Family Welfare Schemes (1950), Urban Revamping Scheme (1984), Sterilization Bed Scheme (1964) and Post Partum Centres (1966). However, the coverage of these schemes was far from satisfactory and limited to mega cities. Moreover, the National Population Policy (2000) and National Health Policy (2002) underline the importance of universal safe delivery coverage. The failure of previous programs led to the establishment of the National Urban Health Mission (NUHM) in 2008. The mission aims to cover 430 cities with more than one million population across the country. The Government of India has allocated a budget of INR (Indian National Rupee) 8600 billion for the first phase of the mission (2008–2012). It aims at addressing the health concerns by facilitating equitable access to available health facilities by rationalizing and strengthening the capacity of the existing health care delivery system [20].

Although the urban health issue has been of long-standing interest to social scientists and public health researchers, majority of the studies have looked upon the urban poor and migrants as distinct subgroups. Moreover, considering the vast socioeconomic and cultural heterogeneity, existing policies and programs face considerable difficulty in identifying the priority sub-groups in urban areas [20]. Against this backdrop, the following questions need to be answered; who is more underserved – the poor or the migrants? What is the progress in the utilization of maternal health care services utilization? What are the factors that associated with maternal health care services utilization among the urban poor and migrants? Another concern is, whether being poor and at the same time migrant leads to a double disadvantage in the utilization of maternal health services?

The present study aims to explore the change in the utilization of safe delivery care during 1992–2006 in urban areas among poor and migrant women, while considering them not as distinct subgroups, but as two interacting groups. Four categories have been generated to understand maternal health care utilization, namely, poor–migrant, poor–non migrant, non poor–migrant, and non poor–non migrant.

## Methods

### Data

The present study utilizes data from the two rounds of the Demographic and Health Survey (DHS), popularly known as the National Family Health Survey (NFHS) carried out during 1992–93 [51] and 2005–06 [52]. The NFHS is a large-scale, multi-round survey conducted in a nationally representative sample of households throughout India. The collaborative efforts of many organizations such as the United States Agency for International Development (USAID), Department for International Development (DFID), United Nations Children’s Fund (UNICEF), United Nations Population Fund (UNFPA) and the Government of India resulted in two rounds of the NFHS. The International Institute for Population Sciences (IIPS), Mumbai, India was appointed as a nodal agency to conduct the surveys. Both rounds of NFHS provided essential state and national level data to monitor health and family welfare programs and policies implemented by the Ministry of Health and Family Welfare and other ministries, national and international agencies.

### Sample Size and Outcome Variable

Both rounds of NFHS recorded total 1,05,807 births from ever married women in the age group, 15–49 years. However, the present study focuses on urban areas, where about 25,545 births were recorded in both rounds of the survey. The first round of NFHS (1992–93) collected information for the last three births in the four years preceding the survey. However, in NFHS-3 (2005–06), information on the utilization of maternal health services included all births to these ever married women in the five years preceding the survey date. In both surveys, detailed information on maternal health care utilization along with other background characteristics were obtained for the most recent birth. In order to increase the robustness of estimates, this study considered only the most recent births and excluded multiple births during the four years preceding the date of survey. Thus, the present study is restricted to 8,410 live births in NFHS 1992–93, and 8,832 in NFHS 2005–06 that occurred in the four years preceding the survey.

This study measured ‘safe delivery’ as per the guidelines developed by the WHO, which included delivery conducted either in a medical institution or home deliveries assisted by doctor/nurse/Lady Health Visitor (LHV)/Auxiliary Nurse Midwife (ANM)/other health professionals [53]. The National Population Policy adopted by the Government of India in 2000 reiterated the government’s commitment to safe motherhood programs within the wider context of reproductive health [54]. Moreover, the national socio-demographic goals for 2010 specified that 80% of all deliveries should take place in institutions and 100% of deliveries should be attended by trained personnel [55].

### Explanatory Variables

Socioeconomic and demographic predictors such as group variables for household wealth and migration status (poor–migrant, poor–non migrant, non poor–migrant, and non poor–non migrant), age of the woman at birth, women’s education, partner’s education, women’s work status, mass media exposure, birth order & interval, status of the last child, religion, caste, and region of residence were included in the study.

The Demographic and Health Survey (DHS) does not collect direct information on income or consumption expenditure of households. However, it has a long history of collecting a whole range of information on housing conditions, consumer durables, and sanitation facilities, which has been widely used as a proxy of household economic status [56]. The DHS wealth index has been widely employed to examine health, population, nutrition, education, and other indicators with respect to economic status [57]–[59]. The World Bank has used the same index for its policy and program recommendations and produced reports for each of the fifty-two countries participating in the DHS program [60].

Similar to other countries’ DHS, a wealth index and its five categories are given in the NFHS 2005–06 dataset. Along the same lines, a modified and comparable wealth index has been constructed for both rounds of the survey on the basis of available information on the ownership of household assets using Principle Component Analysis (PCA) for urban areas [56]. Since the question on assets have also asked to rural households, due attention was paid to include those assets which are appropriate in an urban context while considering the variables for constructing the wealth index (see **Appendix S1**). Each household asset was assigned a weight (factor score) generated and the resulting asset scores were standardized in relation to a normal distribution with a mean of zero and standard deviation of one [61]. The sample was then divided into five equal quintiles categorized as poorest, poorer, middle, richer, and richest [52]. However, to check the internal consistency, that is, how closely all selected household and consumer durable assets considered for the construction of the wealth index are related, Cronbach α test [62], [63] was applied in both rounds of NFHS. The test shows α value for both rounds of NFHS above 0.7 (for NFHS 1992–93: 0.780; and NFHS 2005–06: 0.809), indicating reliability in the estimates.

It is worth reiterating that there is no common consensus among researchers in defining poverty status based on wealth index generated using household ownership of assets. For instance, using DHS data from 23 African countries, a study defined ‘urban poor’ as those who do not have electricity in their households, who do not use drinking water that is, piped water in their home or from a public tap, and do not use a private or shared flush toilet [64]. On the other hand, two studies measured health care utilization and defined ‘poorest’ based on the ownership of household assets by the bottom 40% of the wealth quintile [65], [66]. One study from India computed ‘poor’ and defined ‘proportion poor’ according to state level consumption expenditure estimates [67]. However, another study measured poverty taking into consideration education, health and living standard dimensions of the household [68]. In brief, the concept of poverty is multidimensional and there is no standard definition of the poor as it depends exclusively on the approach, objective and design of the study. However, in this study, the bottom 60% of the wealth quintile has been considered poor and the remaining 40% non poor.

Although there are other sources of migration data in India like the Census, Sample Registration System (SRS), and National Sample Survey Organisation (NSSO), but none of these sources provide comparable information on the utilization of maternal health services over time. Since, the information on migration is usually ignored in large scale surveys, NFHS provides scope to estimate migration status. The survey asked each individual woman, “how long have you (woman) been living continuously in the current place” and respondents were given the options to answer, ‘number of years’, ‘always’ and ‘visitor’. This study refers to a woman other than ‘always resident’ of the urban area as a ‘migrant’; however, ‘visitors’ have been excluded from the analysis. The response in ‘years’ of stay has been considered ‘migrant’.

Other explanatory variables were also included, such as mother’s age at birth, categorized into <18 years and 18–19 years of age. The educational level of women and their husbands was defined using years of schooling and they were grouped into illiterate, literate but primary, primary but below high school, middle but below high school, and high school and above. Religion was categorized as Hindu, Muslim and Others. Identification of the castes/tribes was based on the women’s self-reports as Others, Scheduled Castes (SCs), and Scheduled Tribes (STs). Mass media exposure has been assessed by considering how often the respondents read the newspaper, listen to the radio and watch television or cinema. Since the regional variation in the utilization of maternal healthcare was evident [51], [52] attention was paid to adjust the estimates for region of residence. For this purpose, India was divided into six regions of residence based on geographical location and cultural settings [52]. The six regions consist of North (Jammu and Kashmir, Himachal Pradesh, Punjab, Haryana, Rajasthan, Delhi and Uttaranchal), Central (Uttar Pradesh, Madhya Pradesh and Chhattisgarh), East (Bihar, Jharkhand, West Bengal and Odisha), North-East (Arunachal Pradesh, Assam, Manipur, Meghalaya, Mizoram, Nagaland, Sikkim and Tripura), West (Gujarat, Maharashtra and Goa), and South (Andhra Pradesh, Karnataka, Kerala and Tamil Nadu).

### Analytical Approach

Bivariate analyses were carried out to understand the proportion of difference in safe delivery utilization by four combinations of economic and migrant status. Chi-squared statistics were applied to test the bivariate differences and association in the utilization of safe delivery service by selected background characteristics. The nature of sampling design of both rounds of NFHS allows researchers to pool datasets that facilitate an evaluation of change over time [69], [70].

In order to examine the differential in safe delivery care utilization among four compositions of the poor and migrant, binary logistic regression was applied. The binary response (y, utilized safe delivery care or not) for each individual was related to a set of categorical predictors, *X*, and a fixed effect by a logit link function:
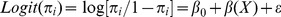



The probability of an individual utilized safe delivery care is *π_i_*. The parameter *β_0_* estimates the log odds of safe delivery care for the reference group, and the parameter *β* estimates with maximum likelihood, the differential log odds of safe delivery care associated with the predictor *X*, as compared to the reference group. *ε* represents the error term in the model. In order to examine the trends in safe delivery care utilization during 1992–2006, an interaction term among the four categories of household wealth and migrant status of women with two time periods was generated. The entire analyses were performed using statistical package, Stata version 10.0 [71]. A pooled binary logistic regression model was fitted confounding for the selected background variables. To test the significance of the interaction among the four study groups with two survey years, the Wald test was performed. This study presents the results of logistic regression models as predicted probabilities with 95% confidence level (CI) in safe delivery progress during the last 15 years to avoid complexities in the interpretation of the results [72]. To estimate the predicted probabilities from logistic regression model, the data analysis was performed using the “SVY” commands available in Stata, which takes care of the design effect of NFHS and allows for adjustments for sampling weights when estimating confidence intervals around the prevalence estimate.

### Ethical Statement

Both waves of the National Family Health Survey (1992–93 and 2005–06) were conducted under the scientific and administrative supervision of the International Institute for Population Sciences (IIPS), Mumbai, India. The institute conducted an independent ethics review of NFHS protocol. Data collection procedures were also monitored and approved by the ORC Macro institutional review board.

## Results

### Descriptive Information of Respondents

**Table 1** presents the percentage distribution of women whose most recent birth occurred during the four years preceding the survey, by selected background characteristics in NFHS 1992 and NFHS 2006. Majority of the women were non poor-migrants (50% in 1992 and 60% in 2006) followed by poor-migrants (32% in 1992 and 22% in 2006) in both rounds of NFHS. Nearly fourth-fifths of the women were in the age group, 20–34. In 1992, two-fifths of the women had no formal education, while in 2006 it was about one-fourth. During both the rounds of the survey, majority of the women were not working, and exposure to any mass media was high. About 29% and 34% women were from the first order birth in 1992 and 2006 respectively, however 32% and 23% belonged to 3+ birth order & >24 months of interval in 1992 and 2006 respectively. Majority were Hindu and belonged to other caste groups in both rounds of NFHS. In 1992 and 2006, about one-fourth of the women were residing in the southern region, followed by the central and western regions.

**Table 1:** Percentage distribution of women who had given their last birth during the last four years preceding the survey, urban India, NFHS 1992–2006.

Backgroundcharacteristics	NFHS 1992–93		NFHS2005–06	
	%	*n*	%	*n*
**Group variable (household wealth and migration status)**				
Poor-migrant	31.5	2413	22.1	1880
Poor-non migrant	8.3	1384	5.0	749
Non poor-migrant	50.2	3842	59.8	5088
Non poor-non migrant	10.1	770	13.1	1115
**Women’s age at birth**				
Below 19	15.3	1285	13.4	1183
20–34	80.1	6738	83.1	7340
35 and above	4.6	387	3.5	309
**Women’s education**				
Illiterate	37.5	3150	25.5	2256
Literate but below primary	6.7	663	5.3	568
Primary but below middle	15.3	1289	16.4	1447
Middle but below high school	11.9	998	15.6	1380
High school and above	28.7	2310	37.2	3182
**Husband’s education**				
Illiterate	19.7	1657	15.5	1370
Literate but below primary	7.2	607	5.2	461
Primary but below middle	15.3	1291	14.5	1282
Middle but below high school	13.7	1156	17.0	1501
High school and above	44.0	3699	47.8	4219
**Women’s work status**				
Not working	85.0	7148	83.4	7365
Work at home	5.0	423	6.3	555
Work away from home	10.0	839	10.3	912
**Mass media exposure**				
No exposure	23.0	1931	10.4	916
Any exposure	77.0	6479	89.6	7916
**Birth order & interval**				
Birth order 1	28.9	2433	33.9	2996
Birth order 2 & interval < = 24	8.5	713	10.0	887
Birth order 2 & interval >24	19.4	1634	24.2	2135
Birth order 3+ & interval < = 24	11.7	983	9.0	798
Birth order 3+ & interval >24	31.5	2648	22.8	2015
**Status of children**				
Wanted	74.4	6252	79.6	7025
Unwanted	25.6	2153	20.4	1806
**Religion**				
Hindu	71.5	6013	73.3	6465
Muslim	21.3	21.3	21.1	1863
Christian/Sikhs/Others	7.2	607	5.6	498
**Castes/tribes**				
Others	86.9	7306	78.6	6820
Schedule Castes	9.6	805	18.1	1571
Schedule Tribes	3.6	299	3.3	285
**Region of residence**				
North	13.1	1098	14.5	1277
Central	23.0	1933	22.8	2012
East	16.0	1343	15.6	1382
Northeast	2.0	167	2.3	204
West	21.1	1776	21.4	1890
South	24.9	2093	23.4	2066
**Total**	**100**	**8410**	**100**	**8832**

Note: Total sample ‘*n*’ may not equal for all variables due to some missing cases.

### Differentials in Safe Delivery Care Utilization

The bivariate differentials in the utilization of safe delivery care during 1992 and 2006 by selected background characteristics are demonstrated in **Table 2**. Only half of the poor-migrant women utilized safe delivery in both rounds of NFHS; however, it was highest among non poor-non migrant women in 1992 (86%) and 2006 (92%). Utilization of safe delivery care in both rounds of NFHS was more than two-thirds (65%) among poor-non migrant women. In both the surveys, the highest users of safe delivery were women in the age group 20–34, and women whose husbands had completed high school education and above. About 42% of illiterate women utilized safe delivery in 1992, and it increased to 48% in 2006. Utilization of safe delivery was lowest among women who worked at home –59% in 1992 and 72% in 2006. Nearly 75% of women with any exposure of mass media had utilized safe delivery in 1992 and it was 81% in 2006. Safe delivery care declined with increase in birth order and interval in both rounds of NFHS.

**Table 2:** Percentage of women who utilized safe delivery care by selected background characteristics, urban India, NFHS 1992–2006.

Background characteristics	NFHS 1992–93	NFHS 2005–06
**Group variable (household wealth and migration status)**	[545.51]***	[1128.65]***
Poor-migrant	49.7	49.5
Poor-non migrant	65.8	66.8
Non poor-migrant	74.4	84.5
Non poor-non migrant	85.5	91.8
**Women’s age at birth**	[65.52]***	[92.46]***
Below 19	64.0	72.4
20–34	68.7	78.6
35 and above	49.9	57.3
**Women’s education**	[1735.91]***	[1725.02]***
Illiterate	41.5	48.0
Literate but below primary	67.5	71.6
Primary but below middle	73.0	76.3
Middle but below high school	78.8	84.1
High school and above	92.7	95.1
**Husband’s education**	[1035.78]***	[1052.90]***
Illiterate	39.0	47.8
Literate but below primary	59.1	65.2
Primary but below middle	60.5	73.1
Middle but below high school	70.5	76.9
High school and above	82.4	89.0
**Women’s work status**	[17.17]***	[20.50]***
Not working	67.9	77.9
Work at home	58.6	71.7
Work away from home	65.2	73.0
**Mass media exposure**	[869.73]***	[605.74]***
No exposure	39.5	44.7
Any exposure	75.4	80.8
**Birth order & interval**	[459.57]***	[804.45]***
Birth order 1	78.9	88.1
Birth order 2 & interval < = 24	70.2	78.7
Birth order 2 & interval >24	75.7	84.6
Birth order 3+ & interval < = 24	55.1	54.3
Birth order 3+ & interval >24	54.7	60.9
**Status of children**	[12.50]***	[103.86]***
Wanted	68.2	79.3
Unwanted	64.1	68.0
**Religion**	[174.70]***	[197.94]***
Hindu	69.3	79.3
Muslim	55.3	65.9
Christian/Sikhs/Others	80.4	90.2
**Castes/tribes**	[172.90]***	[102.56]***
Others	69.8	79.4
Schedule Castes	48.9	69.2
Schedule Tribes	52.5	64.2
**Region of residence**	[730.27]***	[873.84]***
North	54.2	74.5
Central	50.6	57.2
East	61.1	71.4
Northeast	63.1	73.0
West	76.8	87.8
South	85.2	92.3
**Total**	**67.2**	**77.0**

Note: Figures in parentheses are the χ^2^ statistics; χ^2^ test applied for each variable.

Levels of significance:

*** *p*<0.01.

More women from Christian/Sikhs/Other religions utilized safe delivery in 1992 (80%) and 2006 (90%). However, in both survey rounds, safe delivery was least utilized by Muslim women (55% in 1992 and 66% in 2006). Caste wise safe delivery care revealed that the lowest utilization of safe delivery in 1992 was by Scheduled Caste women (49%), while in 2006 it was lowest among Scheduled Tribe women (64%). There were differences in safe delivery by region of residence in both rounds of survey, and showed that utilization of safe delivery was lower among women residing in the central region (51% in 1992 and 57% in 2006), while it was higher among women from the southern region (85% in 1992 and 92% in 2006).

### Progress in Safe Delivery Care Utilization

In order to examine the magnitude of change in the utilization of safe delivery care among women from the four categories of wealth and migrant status in urban India over the period 1992–2006, the binary logistic regression model was used after pooling two rounds of NFHS data (**Table 3**). Unadjusted and adjusted predicted probabilities from the regression analysis considering selected background variables were applied to assess the trends in safe delivery care utilization. The additional two-way interaction among four categories of wealth and migration status with two survey periods was statistically significant, which demonstrated that over the period, safe delivery care with respect to wealth and migration status has changed.

**Table 3:** Predicted probability (95% Confidence Interval) of safe delivery care utilization from the logistic regression analysis for interaction between economic and migrant status with time, urban India, NFHS 1992–2006.

Interaction effect	Unadjusted Predicted Probability	95% CI	Adjusted Predicted Probability^¶^	95% CI
**1992–93**				
Poor-migrant	0.497	[0.472–0.520]	0.502	[0.474–0.529]
Poor-non migrant	0.658	[0.612–0.701]	0.685	[0.633–0.732]
Non poor-migrant	0.744	[0.728–0.759]	0.778	[0.761–0.794]
Non poor-non migrant	0.855	(0.824–0.880]	0.871	[0.839–0.897]
**2005–06**				
Poor-migrant	0.495	[0.466–0.522]	0.476	[0.453–0.517]
Poor-non migrant	0.668	[0.600–0.710]	0.689	[0.581–0.706]
Non poor-migrant	0.845	[0.832–0.856]	0.894	[0.882–0.904]
Non poor-non migrant	0.918	[0.897–0.935]	0.936	[0.917–0.950]
**Change 1992–2006**				
Poor-migrant	−0.002		−0.026	
Poor-non migrant	0.010		0.003	
Non poor-migrant	0.101		0.116	
Non poor-non migrant	0.063		0.065	

Note: **^¶^**Predicted probabilities adjusted for mother’s age at birth, women’s education, husband’s education, work status, mass media exposure, birth order & interval, status of children, religion, caste, and region of residence.

All the predicted probabilities were significantly different at p<0.01 indicates the acceptance of alternative hypothesis in Wald test.

The adjusted predicted probability shows a decline in safe delivery care among poor-migrant women during 1992–2006. The probability of safe delivery care utilization declined from 0.502 (95% CI = 0.474–0.529) in 1992 to 0.476 (95% CI = 0.453–0.517) among poor-migrant women. Over the same period, probability of safe delivery among poor-non migrant women increased from 0.685 (95% CI = 0.633–0.732) in 1992 to 0.689 (95% CI = 0.581–0.706) in 2006. However, in the last 15 years, an increase of 11 and 7 points in safe delivery care utilization was evident among non poor-migrant and non poor-non migrant women in urban areas.

### Factors Associated with Safe Delivery Care

**Table 4** demonstrates the results of the multivariate analyses of the utilization of safe delivery care. Women’s age at birth, women’s education, husband’s education, mass media exposure, birth order and interval, religion, caste and region of residence significantly determine safe delivery care in urban areas, particularly among poor-migrants. The likelihood of safe delivery was higher among poor-migrant women aged 20–34 years compared to women who were below 19 years of age (OR = 1.544; 95% CI = 1.037–2.300). The effect of education on safe delivery care utilization was evident among poor-migrant and non poor-migrant women. The odds of safe delivery among poor-migrant and non-poor migrant who had completed high school and above were nearly three (95% CI = 1.464–7.138) and six (95% CI = 4.147–8.552) times higher respectively than among illiterate women. The likelihood of utilizing safe delivery care increased with the level of husband’s education among poor-migrant women. The odds of safe delivery were found to be lower (OR = 0.598; 95% CI = 0.396–0.902) among poor-migrant women who worked away from home at the time of survey compared with women who were not working. The probability of safe delivery care utilization was found to be higher among those women who had exposure to mass media, than among women who had no exposure to mass media. Poor-migrant women who had exposure to mass media were more likely to utilize safe delivery care compared to women who did not have any exposure to mass media (OR = 1.932; 95% CI = 1.311–2.665).

**Table 4:** Odds ratios (with 95% Confidence Interval) estimated from the binary logistic regression models showing factors associated with safe delivery care by group variable between household wealth and migration status of women, urban India, NFHS 2005–06.

	Poor-migrant		Poor-non migrant		Non poor-migrant		Non poor-non migrant	
Covariates	Odds ratio	95% CI	Odds ratio	95% CI	Odds ratio	95% CI	Odds ratio	95% CI
**Women’s age at birth**								
Below 19 (ref.)	1.000		1.000		1.000		1.000	
20–34	1.544**	[1.037–2.300]	2.145	[0.819–5.616]	2.197***	[1.547–3.120]	0.703	[0.303–1.631]
35 and above	1.410	[0.712–2.793]	6.183**	[1.408–17.148]	1.717	[0.843–3.497]	0.281	[0.040–1.968]
**Women’s education**								
Illiterate (ref.)	1.000		1.000		1.000		1.000	
Literate but below primary	2.080**	[1.308–3.309]	1.957	[0.826–4.634]	1.477	[0.903–2.418]	0.466	[0.138–1.567]
Primary but below middle	1.770**	[1.226–2.553]	3.221**	[1.321–6.850]	1.457**	[1.074–1.976]	1.278	[0.496–3.295]
Middle but below high school	1.662**	[1.053–2.624]	0.949	[0.306–2.942]	2.490***	[1.773–3.496]	1.572	[0.596–4.148]
High school and above	3.233***	[1.464–7.138]	2.475	[0.669–9.158]	5.955***	[4.147–8.552]	7.197***	[2.452–17.126]
**Husband’s education**								
Illiterate (ref.)	1.000		1.000		1.000		1.000	
Literate but below primary	1.556*	[0.976–2.480]	1.222	[0.444–3.361]	0.934	[0.554–1.577]	2.267	[0.345–14.900]
Primary but below middle	1.802**	[1.252–2.592]	3.359**	[1.243–7.080]	1.077	[0.711–1.631]	2.018	[0.784–5.195]
Middle but below high school	1.620**	[1.095–2.397]	1.587	[0.611–4.118]	1.037	[0.693–1.554]	1.588	[0.568–4.440]
High school and above	1.493*	[0.936–2.382]	0.980	[0.332–2.886]	1.168	[0.786–1.735]	2.571*	[0.998–6.626]
**Women’s work status**								
Not working (ref.)	1.000		1.000		1.000		1.000	
Work at home	1.138	[0.672–1.928]	0.377	[0.105–1.361]	0.841	[0.547–1.294]	0.738	[0.306–1.781]
Work away from home	0.598**	[0.396–0.902]	0.639	[0.299–1.366]	1.057	[0.684–1.634]	5.899**	[1.913–18.191]
**Mass media exposure**								
No exposure (ref.)	1.000		1.000		1.000		1.000	
Any exposure	1.932***	[1.311–2.665]	3.180**	[1.869–5.471]	1.871***	[1.276–2.744]	1.632	[0.144–18.52]
**Birth order & interval**								
Birth order 1 (ref.)	1.000		1.000		1.000		1.000	
Birth order 2 & interval < = 24	0.455**	[0.265–0.782]	0.629**	[0.193–2.049]	0.496***	[0.332–0.740]	0.430	[0.154–1.202]
Birth order 2 & interval >24	0.659*	[0.420–1.035]	1.045	[0.333–3.280]	0.534***	[0.383–0.745]	0.476	[0.207–1.092]
Birth order 3+ & interval < = 24	0.321***	[0.198–0.520]	0.334*	[0.096–1.166]	0.303***	[0.200–0.459]	0.305**	[0.123–0.756]
Birth order 3+ & interval >24	0.410***	[0.265–0.635]	0.358*	[0.109–1.175]	0.405***	[0.285–0.576]	0.480	[0.198–1.162]
**Status of children**								
Wanted (ref.)	1.000		1.000		1.000		1.000	
Unwanted	1.151	[0.835–1.585]	0.840	[0.438–1.611]	0.937	[0.723–1.215]	0.616	[0.338–1.120]
**Religion**								
Hindu (ref.)	1.000		1.000		1.000		1.000	
Muslim	0.613***	[0.368–0.812]	0.740	[0.320–1.710]	0.754**	[0.577–0.987]	1.844	[0.819–4.150]
Christian/Sikhs/Others	0.967	[0.439–2.133]	1.404	[0.447–4.408]	1.216	[0.669–2.211]	2.395	[0.441–12.999]
**Castes/tribes**								
Others (ref.)	1.000		1.000		1.000		1.000	
Schedule Castes	0.414***	[0.291–0.720]	1.284	[0.610–2.706]	0.784	[0.586–1.049]	2.353*	[0.967–5.722]
Schedule Tribes	0.570*	[0.308–1.056]	0.730	[0.226–2.362]	0.510**	[0.261–0.998]	0.223*	[0.039–1.280]
**Region of residence**								
South (ref.)	1.000		1.000		1.000		1.000	
North	0.138***	[0.089–0.215]	0.052***	[0.011–0.253]	0.281***	[0.197–0.400]	0.095***	[0.040–0.229]
Central	0.124***	[0.088–0.174]	0.050***	[0.023–0.110]	0.164***	[0.117–0.229]	0.059**	[0.025–0.140]
East	0.204***	[0.143–0.291]	0.216***	[0.101–0.462]	0.363***	[0.247–0.534]	0.242*	[0.085–0.690]
Northeast	0.170***	[0.082–0.352]	0.128***	[0.038–0.428]	0.323**	[0.146–0.715]	0.340	[0.061–1.890]
West	0.464***	[0.307–0.702]	0.277**	[0.114–0.674]	0.604**	[0.425–0.860]	0.489	[0.202–1.183]

Level of significance:

*** p<0.01;

** p<0.05;

* p<0.10.

ref.: Reference category.

The effect of birth order and interval appeared to be a significant factor affecting postnatal care utilization among poor-migrant and non poor-migrant women. The odds of utilizing postnatal care was lower among poor-migrant women with third order births with more than 24 months of previous birth interval compared to women with first order births (OR = 0.410, 95% CI = 0.265–0.635). The likelihood of using safe delivery care was lower among poor–migrant women belonging to the Muslim religion (OR = 0.613, 95% CI = 0.368–0.812) compared to women belonging to the Hindu religion. Similarly, the odds of utilizing safe delivery care was found to be less likely among Scheduled Caste women with a poor-migrant status (OR = 0.414, 95% CI = 0.291–0.720) than among women from other castes. The effect of region of residence on safe delivery care utilization was evident across all four categories. In general, the result shows that compared to the southern region, the odds of utilizing safe delivery care were lowest in the central region, followed by the northern region.

## Discussion

After the ICPD conference, India experienced various shifts at the policy level. In 1997, the integration of the Child Survival and Safe Motherhood (CSSM) program (1992–1996) into the broad umbrella of the Reproductive and Child Health Program (RCH) was a major move. Since then, the RCH services continued to be an integral part of the all basic health care services, irrespective of the coverage, be it rural or urban. An important thrust of the RCH program was to encourage deliveries in proper hygienic conditions under the supervision of trained health professionals [54]. However, urban health issues have long been ignored due to the conventional belief that they have an advantage over rural areas and that their “average” health achievement is impressive. But along with increasing urbanization, the growing socio-cultural and economic heterogeneity of the urban population has forced researchers and program personnel in recent years to acknowledge the progress beyond average. This study attempts to understand the trends and differences in the utilization of safe delivery care among four distinct urban population groups: poor-migrant, poor-non migrant, non poor-migrant, non poor-non migrant.

The findings of the present study documented a sharp differential in the utilization of safe delivery care among the four groups of household wealth and migrant status within urban areas in India. Poor-migrant women seemed to be greatly disadvantaged in the utilization of safe delivery care. Barely fifty percent of the poor-migrant women were utilizing safe delivery care, while about nine out of ten non poor-non migrant women had given their last birth in proper hygienic conditions under the supervision of trained health professionals. Results indicate that during the last 14 years, safe delivery care utilization by those other than poor-migrant women has increased. The present study reiterates that urban inequality in the utilization of maternal health services is not merely restricted to the urban poor; rather the position of poor-migrant women has worsened.

Evidence from other countries points to the ‘package’ of obstacles in utilizing health care services among migrants ranging from low social status, low income and education, low level of awareness and poor access to fair credits [73], [74]. A longitudinal study in Indonesia has shown that despite earning a high income, migrants tend to under consume and remit a large amount of earnings to families at the place of origin, which hinders migrants own potential health gains [75]. In addition, the low coverage of health care utilization among poor households could be due to the low priority assigned to health seeking over other basic daily living needs [4]. The underutilization of health services by poor-migrant women can be linked to residential segregation and detached social networks that perpetuate ‘traditional’ beliefs and behaviors and effectively deny access to modern health services [46]. In India, studies have argued that public opinion on poor and unskilled migrants tends to be very hostile; they were often blamed for the overburdened civic amenities and facilities and deteriorating urban environment and sanitation [76] – could adversely affect poor-migrants’ access to health care facilities. Additionally, poor-migrant households were primarily engaged in the informal sector and the travel costs along with waiting and opportunity costs for utilizing health care services were possibly high [77].

Neglecting social determinants of health highlighted by the WHO [78] and failure of the urban health system to provide essential health services to all, has resulted in the poor health status of the marginalized sections of society, especially poor-migrants. There is evidence that areas predominantly occupied by poor-migrants are not notified in official records and in many cases, are outside the purview of civic and health services [79]. In the last two decades several policies and programs have been launched, but they neither focused on identifying the marginalized sections in urban areas nor did they encourage targeted interventions. Even defining the poor in India is an issue that is very sensitive, debatable and with an unfinished agenda, which poses a major hurdle in poverty alleviation efforts. Recent estimates show that during the last two decades, the fall in the extent of absolute poverty was modest in India [80]. It has often argued that schemes designed ‘only for the poor’ are likely to end up as ‘poor schemes’ in design, implementation and monitoring [81].

This study also examines factors associated with safe delivery care among four distinct groups of women by selected background characteristics. Predominantly, safe delivery care was significantly influenced by women’s education, husband’s education, mass-media exposure, birth order & interval, religion, caste and region of residence. Education of women exerts a significant influence on safe delivery care. However, the effect was neither constant across all levels of education nor was it similar for the four categories. Studies from developing countries consistently documented mother’s education as one of the most prominent factors affecting maternal health care utilization, after controlling for other potential confounders [82]–[85]. Education serves as a proxy for information, cognitive skills and values, which uneducated women often lack [86]. Illiterate or less educated women do not have the confidence and capability to make decisions regarding utilization of health services [7]. Moreover, illiterate women are less likely to be aware about the benefits of proper health care services, resulting in lower utilization when compared to women with higher education [87]. Along with women’s education, the findings of this study confirm the significant effect of husband’s education on the utilization of maternity care services among poor-migrant women. This could be attributed to the educated husband’s involvement in the wife’s maternal health care needs. Studies elsewhere have also shown that male involvement in wife’s health needs enhances maternal health care choices and their utilization [88], [89]. Evidences have also established that when husbands agreed on the importance of women’s health care, they addressed it in time [90].

Mass media exposure is significantly associated with the use of safe delivery care. The results show that poor-migrant women who had no exposure of any mass media were the lowest users of safe delivery care. Studies have shown that exposure to mass media promotes health-related behavior including contraceptive use and reproductive preferences [91], [92]. Mass media is an important source for spreading information on the availability and importance of health care services [7]. It could also be used to bring about changes in people’s attitudes towards the utilization of modern medical services [93]. Birth order is negatively associated with the utilization of safe delivery care. Evidence from developing countries indicated that women are significantly more likely to use maternity care services for their first delivery [87], [94]. A study conducted in the six developing countries had also observed that high order women are the least likely to deliver with a health professional [95]. A study performed in a north Indian city among low income groups found that women are more likely to get care for their first delivery than for those that follow [96]. Reasons for this are delivery related factors such as fear of the unknown or excitement over a first child, inexperience in pregnancy and higher health risks for women and their children [89]. However, high parity women accumulated more experience and often felt that they may not need birth assistance [95].

The population composition of India is significantly influenced by religious and social groups, like Muslims and Scheduled Castes. The present study shows that poor-migrant Muslim women were less likely to utilize safe delivery care compared to Hindu women. The prevalence of a similar pattern among caste groups demonstrated lower utilization of delivery care among poor-migrant Scheduled Castes women compared to women from other castes. Previous studies have documented poor health and demographic indicators, along with high inequality in income and education among Muslims and Scheduled Castes in India [96]–[101]. This finding confirms that social group inequality has been persisting in urban areas, particularly among poor-migrants and that there could be lack of access to modern health services among this underserved group.

The considerable regional variation in the utilization of safe delivery care among all four groups can be broadly linked with the north-south dichotomy which has been highlighted by several studies [102]–[104]. India exhibits one of the highest demographic heterogeneities ever experienced at the regional and state levels [50]. This finding adds to the existing evidence that regional differences persist in maternity care utilization among different sub-groups of poor and migrant women in urban areas. Recent studies have documented vast regional variations in the utilization of maternal and child health services [33], [85]. In the last few years, considerable improvement in the utilization of maternal care services was evident in the southern region, while the least change was observed among states in the northern and central regions [33]. In India, about 55% of the total population lived below the poverty line in states covered under the central and eastern regions [105]. These states were characterized by low urbanization, high illiteracy, poor exposure to mass media and low mean age at marriage. On the contrary, the southern region is more socially, economically and demographically advanced than the other regions [102], [106].

The National Rural Health Mission (NRHM) with an objective to raise the overall health status of the rural population has been paying special attention to High Focus States (HFS) selected on the basis of poor socioeconomic, demographic and health indicators, particularly for rural areas [107]. This study proposed to adopt a similar approach under the NUHM and identified High Urban Priority States (HUPS) to formulate a comprehensive health system response towards growing urban health needs in a few regions.

### Conclusion

This study adds to the existing knowledge about the health status in urban India and takes an initial step in exploring the urban health inequality beyond rich-poor differences. The differences in safe delivery care utilization with respect to four categories of economic and migrant status is considerable. The present study identifies two distinct groups in terms of safe delivery care utilization in urban India: one for poor-migrant and one for non poor-non migrants. While poor-migrant women were found to be the most vulnerable, non poor-non migrant women were the highest users of safe delivery care. This study highlights some of the priority areas which could be considered under the second phase (2012–2016) of the NUHM. The ongoing programmatic efforts must start focusing on the poorest of the poor, that is, poor-migrant women. Moreover, specific attention is needed to educate poor-migrant women about the benefits of timely and proper utilization of health services. It is also essential to bring home the importance of safe delivery care utilization to uneducated or less educated husbands of poor-migrant women. Special efforts must be made by Urban Social Health Activists (USHA), under NUHM to reach higher order mothers for timely and appropriate counselling regarding the advantage of availing maternal health services.

An extensive media driven awareness initiative is needed to raise the consciousness towards the utilization of health services among poor-migrant women belonging to Scheduled Castes and the Muslim community in urban areas. Mobile communication, FM radios and other means of electronic media have an impressive reach among the poor-migrants in urban areas, and could therefore be utilized extensively as useful means of disseminating health related knowledge. More importantly, continuous evaluation is imperative that will help to examine the real progress in the utilization of health services in urban areas, particularly among target groups like poor-migrants, and accordingly, priorities should be set for future urban health policies. It is also important to set aside the misconceptions that have prevented the utilization of health care services by the urban population impressive in order to evaluate real progress.

### Study Limitations and Further Research

The limitations of this study must be understood in the light of the results. While the quality of DHS data is quite high, information on the use of safe delivery is subject to recall errors. This study has attempted to minimize these errors while considering most recent births that occurred in the four years preceding the survey. The present study attempts to examine the trends and factors of safe delivery considering selected background variables, while measuring clinical quality, service delivery environment and quality control dimensions have not been considered. Secondly, since data on direct income were not collected in DHS, this study defined the economic status of women on the basis of ownership of household and consumer assets. Considering the streams of migration while examining the utilization of maternal health care services can be an important avenue for future public health research. In order to understand the health needs of the poor-migrants in urban areas, there has to be further research on the behavioural aspects, service delivery issues and qualitative barriers that restrict maternal health services utilization. Another area of future research could be the utilization of health services among poor-migrants by their changing economic status over time.

## Supporting Information

Appendix S1 **List of variables used for constructing wealth index for urban India, NFHS 1992 & 2006.**
(DOC)
