# Supplementary material for: Examining the Effect of Household Wealth and Migration Status on Safe Delivery Care in Urban India, 1992–2006
Source: PLoS One. 2012 Sep 7;7(9):e44901. doi: 10.1371/journal.pone.0044901 (PMC3436793; doi:10.1371/journal.pone.0044901)
Supplement: Appendix S1 — List of variables used for constructing wealth index for urban India, NFHS 1992 & 2006. (DOC) [file pone.0044901.s001.doc]

**Appendix S1:** List of variables used for constructing wealth index for urban India, NFHS 1992-93 & 2005-06

| **List of variables** | **NFHS 1992-93** | **NFHS 2005-06** |
| --- | --- | --- |
| **Household goods** |  |  |
| Radio | NA | C |
| Watch | C | C |
| Pressure cooker | NA | C |
| Electric fan | C | C |
| Sewing machine | C | C |
| Television (black and white) | C | C |
| Television (colour) | NA | C |
| Refrigerator | C | C |
| Mattress | NA | C |
| Chair | NA | C |
| Cot/bed | NA | C |
| Table | NA | C |
| **Household condition and sanitation** |  |  |
| *Pucca* house | C | NC |
| Semi *pucca* house | C | NC |
| *Kaccha* house | C | NC |
| Floor material (natural/rudimentary or finished) | NA | C |
| Wall material (natural/rudimentary or finished) | NA | C |
| Roof material (natural/rudimentary or finished) | NA | C |
| No window | NA | C |
| Window without cover | NA | C |
| Window with cover | NA | C |
| Ownership of house | NA | C |
| 2 Persons per room | C | C |
| 2 to 4 persons per room | C | C |
| More than 4 persons per room | C | C |
| Has separate kitchen | C | C |
| **Household fuel and water sources** |  |  |
| Own arrangement of drinking water | C | C |
| Drinking water from public tap and small tank | C | C |
| Other sources of drinking water | C | C |
| Fuel type | C | C |
| No toilet | C | C |
| Pit toilet | C | C |
| Flush toilet | C | C |
| Water pumps | C | C |
| **Means of transportation** |  |  |
| Bicycle | C | C |
| Motorcycle | C | C |
| Car | C | C |
| **Means of communication** |  |  |
| Landline phone | NA | C |
| Mobile phone | NA | C |
| Computer | NA | C |
| **Others** |  |  |
| Bank account/post office account | NA | C |
| Electricity | C | C |
| **Agricultural related** |  |  |
| Any land | NC | NC |
| Marginal land | NC | NC |
| Any irrigated land | NC | NC |
| Live stocks | NC | NC |
| Thresher | NC | NC |
| Tractors | NC | NC |
| Note: NFHS, National Family Health Survey; C, considered; NC, not considered; NA, not available. | | |
